# 

**Published:** 1916-12

**Authors:** 


### Contents

Event and Comment........................................ 531

Modeling Compound *vs.* Plaster of Paris.................. 535

Mercury in Pyorrhea........................................ 566

Indents. ................................................... 571

Obituary ................................................... 588

Bibliography ............................................... 591

Injection Treatment of Neuritis with Hot Saline Solution.... 592

